# Comparison of optimal performance at 300 keV of three direct electron detectors for use in low dose electron microscopy

**DOI:** 10.1016/j.ultramic.2014.08.002

**Published:** 2014-12

**Authors:** G. McMullan, A.R. Faruqi, D. Clare, R. Henderson

**Affiliations:** a MRC Laboratory of Molecular Biology, Francis Crick Avenue, Cambridge CB2 0QH, United Kingdom; b Crystallography and Institute of Structural and Molecular Biology, Birkbeck College, University of London, Malet Street, London WC1E 7HX, United Kingdom

**Keywords:** DQE, MTF, CMOS

## Abstract

Low dose electron imaging applications such as electron cryo-microscopy are now benefitting from the improved performance and flexibility of recently introduced electron imaging detectors in which electrons are directly incident on backthinned CMOS sensors. There are currently three commercially available detectors of this type: the Direct Electron DE-20, the FEI Falcon II and the Gatan K2 Summit. These have different characteristics and so it is important to compare their imaging properties carefully with a view to optimise how each is used. Results at 300 keV for both the modulation transfer function (MTF) and the detective quantum efficiency (DQE) are presented. Of these, the DQE is the most important in the study of radiation sensitive samples where detector performance is crucial. We find that all three detectors have a better DQE than film. The K2 Summit has the best DQE at low spatial frequencies but with increasing spatial frequency its DQE falls below that of the Falcon II.

## Highlights

• Three direct electron detectors offer better DQE than film at 300 keV.
• Recorded 300 keV electron events on the detectors have very similar Landau distributions.
• The Gatan K2 Summit detector has the highest DQE at low spatial frequency.
• The FEI Falcon II detector has the highest DQE beyond one half the Nyquist frequency.
• The Direct Electron DE-20 detector has the fastest data acquisition rate.

## 1 Introduction

Electron microscope images were originally recorded on photographic film and more recently electronically using detectors based on phosphor/fibre-optic CCD technology. These work well for electron energies in the 80–120 keV range but at higher electron energies their performance drops. Higher electron energies are necessary with thicker samples and advantageous in looking at insulators, such as ice embedded biological samples, due to the reduced sensitivity to sample charging. The shorter wavelength also results in improved electron optics and simpler interpretation of the resulting images.

The decrease in imaging performance of traditional detectors at higher electron energies can be traced to reduction in the interaction cross-section with increased energy. Higher energy electrons deposit a lower, and more variable, amount of energy at their initial point of incidence. Their subsequent path has a far greater range leading to the appearance of tracks in the detector and contributions from where electrons backscatter either from deeper within the substrate of the detector or from the surrounding housing. The lower, and more variable, initial signal combined with the addition of backscattering events that contribute to the noise results in a lower signal-to-noise even near zero spatial frequency. At higher spatial frequencies the performance is further degraded by the stochastic nature of electron trajectories and the fact that the rate of energy loss by an electron increases as the electron slows down.

Detector performance is of particular importance in the study of radiation sensitive samples such as in electron cryo-microscopy (cryoEM), where the signal-to-noise ratio in images is inherently poor due to the limited number of electrons that can be used before radiation damage is too great. The amount of additional noise added by a detector is measured by its detective quantum efficiency (DQE) which is defined [1] as the square of the ratio of the output signal-to-noise, ${\mathrm{SNR}}_{o}$, to that of the input, ${\mathrm{SNR}}_{i}$, i.e.,

(1) $$\mathrm{DQE}={\mathrm{SNR}}_{o}^{2}/{\mathrm{SNR}}_{i}^{2}.$$

Ideally a detector would not add any noise and so have a DQE of 1 but all real detectors have values less than 1.

Direct detection of electrons using backthinned monolithic active pixel sensors (MAPS) has emerged as the most promising technology with which to produce detectors with high DQE at higher incident electron energies [2], [3], [4]. MAPS detectors are fabricated in silicon using industry standard CMOS imaging technology that enables the manufacture of uniform large format (≥4k×4k) pixel sensors. Their potential for use as high DQE detectors is reflected in the high signal to noise with which they are capable of detecting individual incident 300 keV electrons. They are susceptible to radiation damage and despite an increase in radiation hardness with smaller dimension fabrication technology, any practical detector must make use of radiation-hard design techniques.

The range of a 300 keV electron in silicon can exceed 300μm and so it is not practical to limit the signal from an individual incident electron to a single pixel. MAPS detectors can however have most of their support matrix removed so that a functioning detector can consist of only a thin membrane (≤50μm) through which incident 300 keV electrons can easily pass. In order to maximise the benefit of this process it is also important to mount a detector carefully to prevent transmitted electrons from scattering back into the detector from the camera housing.

MAPS detectors are capable of high readout speeds and this can be used to ameliorate the effects of radiation damage by limiting the contribution in any frame from increased leakage current associated with radiation damage. The combination of high DQE and high readout speed also gives greater flexibility in imaging. For example, images can be recorded as dose-fractionated movies from which the optimal exposure can be selected during image processing, long after the specimen has been removed from the microscope. The combination of high sensitivity and readout speed makes it possible to use a counting mode in which a final image is reconstructed from processed sub-images of individual electron events [5]. This enables the intrinsic variability in the signal left by an incident high energy electron to be removed and so achieve a higher DQE, at least at low spatial frequency. It is also possible to infer the initial point of incidence of an electron to sub-pixel resolution (super-resolution mode) and so obtain information beyond the traditional Nyquist frequency limit.

In this paper we present MTF and DQE measurements of the three currently available backthinned MAPS detectors that offer improvements over photographic film in terms of DQE at 300 keV namely: the Direct Electron DE-20^1^; the FEI Falcon II^2^; and the Gatan K2 Summit.^3^ It is possible to record dose-fractioned movies with all three detectors and in principle operate any of the detectors in a counting mode. In this paper only counting mode results obtained using the K2 Summit will be presented.

## 2 Methods

The Falcon II and K2 Summit detectors were both installed on a FEI Titan Krios at the MRC Laboratory of Molecular Biology (MRC-LMB) in Cambridge while the DE-20 detector was installed on a FEI Polara G2 at Birkbeck College in London. Both microscopes were fitted with Gatan energy filters (GIF Quantum on the Titan Krios and GIF 2002 on the G2 Polara). The Falcon II and DE-20 detectors were both positioned before their respective energy filters while the K2 Summit was positioned after the energy filter. The energy filter was operated without an energy slit and carefully tuned before any measurements were taken.

In order to compare the detectors on different microscopes accurately the exposure meter on each microscope was first calibrated. To do this the exposure meter readings were compared at a series of different beam currents obtained by altering the spot size. The beam current was measured using the drift tube of the energy filter as a Faraday cup. The same SEM (scanning electron microscopy) probe current meter^4^ was used to measure the beam currents on both microscopes. The accuracy of this meter was confirmed to better than 1% in the range of interest using a calibrated laboratory voltage standard and combinations of high precision 100MΩ resistors. For a given spot size a small diameter beam was positioned so that with the energy filter set to 300 keV the beam could be seen to pass entirely through the energy filter. The beam current was then measured by setting the energy filter voltage to zero so that the entire beam hits the drift tube. The corresponding microscope exposure meter reading was obtained by lowering the flu-screen. As the beam current can vary with changes in either the condenser or objective lens strengths, care was taken not to alter the beam settings during a measurement. After each current measurement the energy filter voltage was set back to 300 keV in order to verify that the entire beam once again passed through the energy filter. On both microscopes the exposure meter readings obtained in this way could accurately be described as a linear function of the corresponding probe current meter readings, though with different slopes and offsets. The calibrations of both the Krios and the Polara are given in supplementary information. Allowing for the uncertainty in the exposure meter reading the total current in a beam could be measured to within 3%.

The number of electrons incident per second on a pixel was calculated from the measured current in a defined circular beam contained entirely on the detector. To minimise Fresnel effects at the edge of the beam, a selected area aperture was used to define the beam on the detector and the lowest possible magnification used. The microscope exposure meter on the Krios microscope did not register currents below 42 pA. To measure lower currents a beam with greater than 42 pA was first set and the required, incident rate for electrons on the detector obtained by increasing the microscope magnification. Unlike the case with changes in either the objective or condenser lens the measured beam current is not sensitive to changes in magnification resulting from the diffraction, intermediate or projector lenses, provided the entire beam remains on the flu-screen (which provides the input to the exposure meter). The relative magnifications between the different microscope settings were calibrated using images at the different magnifications centred on the same area of a sample.

Backthinning a detector by itself does not guarantee improved performance from a detector. In particular, backscattering from the silicon substrate of a detector may simply be replaced by backscattering from the aluminium alloy that typically lines a camera housing. As the housing is further away the backscattering contribution will be moved to lower spatial frequency. The simplest and most effective way to reduce the amount of backscatter is to increase the distance between the backthinned detector and its housing. For large detectors such as the Falcon II it is not easy to find sufficient space under the detector and additional steps such as replacing any aluminium by lighter elements such as beryllium, boron or carbon are needed. The amount of backscatter from the housing can be measured by taking a series of images in which the edge of a large selected area aperture is scanned across the detector. Electrons passing through a detector undergo many collisions and so by the time they leave the detector their probability of being backscattered to the detector by the housing can be taken to be uniform. The presence of backscatter shows up in both the illuminated and shadow areas as an additional contribution that is proportional to the area of the detector being illuminated. With the DE-20 this contribution was found to be 5% while for both the K2 and Falcon II detectors it was less than 2%.

The DQE of detectors is in general dependent on the dose rate and for this work we have attempted to use a dose rate that was optimised for each detector. For the K2 Summit this meant using as few electrons per frame as possible. A value of 1.1 e/pixel/s, or 1 electron per every 360 pixels in an individual frame, was used. For both the DE-20 and the FEI Falcon II the dose rate was chosen so that the peak in the histogram from an image of an individual frame was positioned at approximation 1/3 of the detector׳s dynamic range. This criterion meant that the DE-20 and the Falcon II were operated with 4 and 3 electrons per pixel per frame, respectively. As the DE-20 was set to run at 25 fps its dose rate, i.e., 100 e/pixel/s, was nearly twice that of the Falcon II operating at 18 fps, i.e., 54 e/pixel/s. In practice the high signal-to-noise seen in both the DE-20 and the Falcon II means that much lower dose rates can also be used without significantly degrading the DQE.

The MTF and DQE were measured using the procedure described in [4]. The MTF was measured using the shadow image of a platinum rod. In the Polara this consisted of a 2 mm diameter rod inserted at the pointer position. This was not possible with the Krios but as the microscope was fitted with a film mechanism a modified film holder was used to support a 1 mm rod. This was positioned manually using a syringe to pressurise the film insertion mechanism. The accuracy of the MTF obtained from the shadow image of a sharp edge depends on the quality of the edge. Only straight, blemish free sections from the images were used. For measuring the MTF of the K2 Summit extra care was needed due to its intrinsically high MTF, small pixel size and the additional ×6 magnification from the energy filter. The quality of an edge was verified by examining the difference between the original image and the simulated edge image blurred by the fitted MTF. As in [4] a sum of Gaussian functions is used as a convenient analytical fit. This type of fitting is known to be an ill-posed problem and the validity of any fit was always checked with results from direct numerical differentiation of the measured edge spread function.

The MTF of the Falcon II was also calculated using the noise power method [1]. In this the signal from an incident electron is described by a circularly symmetric point spread function, PSF, and the MTF obtained from the Fourier transform of this PSF. In this work the PSF is expanded as a normalised sum of Gaussian functions in which the weights and length parameters are obtained by fitting to the measured noise power spectra (see Appendix A). The noise power method typically over estimates the high frequency MTF, since the noise power spectra reflect the stochastic response to individual electrons rather than the averaged response as measured by the MTF [6], [7].

The gain of a counting detector, such as the K2 Summit, decreases as the probability of two or more electrons being recorded in or around a pixel increases (see Appendix B). The resulting non-linearity will in general need to be corrected for, especially in high contrast images such as that of the sharp edge used to measure the MTF. To avoid this complication, the MTF of the K2 Summit was measured with a very low dose rate (~1 e/pixel/s) that required using long exposures (~300 s).

## 3 Results

**Fig. 1:**
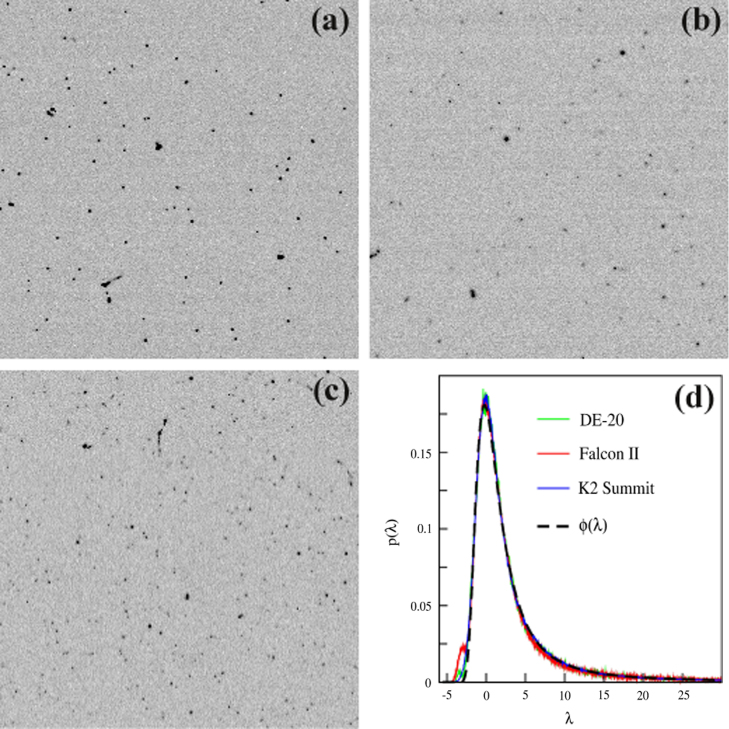
Randomly chosen 256×256 areas from single frames showing individual 300 keV electron events as recorded on the (a) DE-20, (b) Falcon II and (c) K2 Summit detectors. The images are normalised so that the RMS background noise has a value of 1. In (d) the Landau distribution, ϕ(λ), is compared with the measured probability distributions for events as a function of integrated event signal, Δ, in the three detectors. The measured distributions have been scaled by the fitted width parameter, *ξ*, and plotted using $\lambda =[\Delta -(\Delta_{\mathrm{mp}}-\xi \lambda_{0})]/\xi$ in which $\lambda_{0}=-0.2228$ is the position of the maximum of ϕ(λ), $\Delta_{\mathrm{mp}}$ is the position of the most probable value. The measured ratios of $\Delta_{\mathrm{mp}}$ to the RMS background noise for the DE-20, Falcon II and K2 Summit are 49.6, 30.6 and 19.6 respectively, while the corresponding ratios of $\Delta_{\mathrm{mp}}$ to *ξ* are 4.8, 5.3, and 5.4.

The physical properties of the three detectors are summarised in Table 1. The high sensitivity of MAPS detectors to individual 300 keV electrons is illustrated in Fig. 1(a–c). These show images of single electron events as recorded in single frames on the detectors. Single frame output is not currently supported on the K2 Summit and in order to obtain this image the data-stream from the camera was intercepted and decoded. The actual frames and the parts of them that are shown were chosen randomly. In order to compare the detectors the signals have been scaled so that the RMS readout noise in each pixel is 1. The intrinsic variability in the events can be clearly seen as well as the presence of the occasional electron track.

**Table 1:** Physical properties of the detectors.

Detector	Sensor size	Pixel size (μm)	Readout speed (fps)
DE-20	5120×3840	6.4	25^a^
Falcon-II	4096×4096	14.0	18
K2 Summit	3838×3710	5.0	400

a The DE-20 is capable of operating up to 32.5 fps.

In high energy physics the intrinsic variability of the energy loss by a charged particle passing through a thin absorber is known as straggling and the distribution of energy loss usually fitted to a Landau distribution, ϕ(λ) [8]. In Fig. 1d the measured probability distributions for integrated signal of individual events as a function of signal are compared with the Landau distribution. The distributions were calculated from images in which individual incident electrons could easily be resolved. A threshold of 4 times the average readout noise was used to identify a seed pixel of an event. Having identified a seed pixel all contiguous pixels also above the threshold were used to define an event and the total signal, Δ, for an event obtained by summing the contributions from pixels both in the event and from within a radius of 2 pixels around the event. The probability distribution, p(Δ), for events was then fitted to a scaled Landau distribution ϕ(λ)/ξ in which $\lambda =[\Delta -(\Delta_{\mathrm{mp}}-\xi \lambda_{0})]/\xi$, $\lambda_{0}=-0.2228$ is the position of the maximum of ϕ(λ), *ξ* is a fitted width parameter and $\Delta_{\mathrm{mp}}$ is the position of the most probable value of p(Δ). As can be seen in Fig. 1d, the measured distributions fit the functional form of Landau distribution very well. The small pedestal in the measured distributions visible at low Δ is due to the erroneous inclusion of noise events and can be removed using a higher threshold. The absolute scale of Δ in terms of energy was not calibrated but the ratio of $\Delta_{\mathrm{mp}}$ to the noise gives a measure of the signal-to-noise in the detector. The values of this ratio are given in the caption of Fig. 1. The theoretical mean of a Landau distribution is undefined due to its infinitely long tail. In reality there is an upper limit on Δ and using the measured range to set the limits gives a mean value for Δ that is essentially twice the most probable value, $\Delta_{\mathrm{mp}}$. From the values of $\Delta_{\mathrm{mp}}$, given in the caption of Fig. 1, the ratios of the mean signal to the readout noise in a pixel are 99, 61 and 39 for DE-20, Falcon II and K2, respectively.

In the absence of readout noise the value of DQE(0) can be calculated from the first two moments of p(Δ) using

(2) $$\mathrm{DQE}(0)={\left(\int p(\Delta )\Delta \;d\Delta \right)}^{2}/\int p(\Delta )\Delta^{2}\;d\Delta .$$

If the distributions, p(Δ), for the detectors were actually the same the detectors would be expected to have the same DQE(0). Using Eq. (2) and the measured distributions result in values for DQE(0) between 0.34 and 0.48. The actual values are however unreliable as they are very sensitive to systematic errors in calculating Δ and to small differences (especially at high Δ) in p(Δ).

**Fig. 2:**
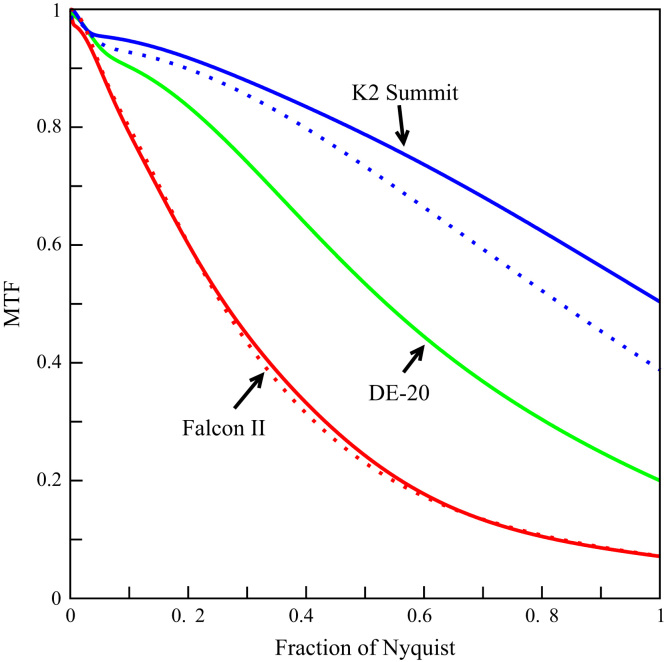
Measured MTF as a function of spatial frequency. The solid lines are for the DE-20 (green), Falcon II (red), and K2 Summit in super-resolution mode (blue). The dotted blue line is the corresponding K2 Summit result in normal resolution mode. The dotted red line is the MTF obtained for the Falcon II via the noise power spectra method.

The measured MTF as a function of spatial frequency for the three detectors is shown in Fig. 2. The corresponding edge spread functions and Gaussian expansion fits are given as supplementary data. The MTF of the K2 Summit was obtained both in normal and super-resolution (but plotted only out to the physical Nyquist frequency). Using super-resolution leads to a significant improvement in the MTF but the actual enhancement is less than that expected from the reduction in the pixel modulation factor, i.e., 2sin(πx/2)/sin(πx/4) that would be expected if the hardware centroiding algorithm used by the K2 worked perfectly. The results for the Falcon II MTF calculated using both the edge and noise power spectra methods agree very well. While the MTF of the Falcon II is lowest, the agreement between these two methods for calculating the MTF indicates that to a first approximation its response to incident electrons can be described by a simple point spread function.

**Fig. 3:**
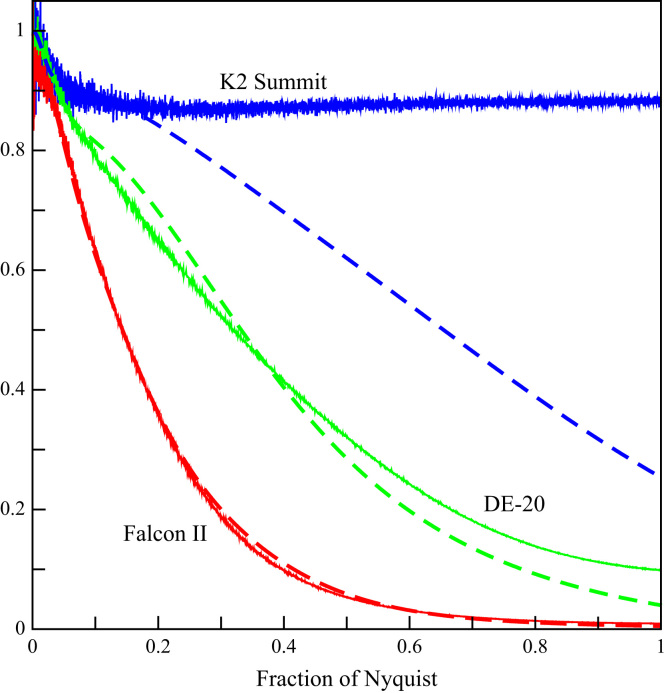
Comparison of the behaviour as a function of spatial frequency of noise power spectra (solid) and ${\mathrm{MTF}}^{2}$ (dashed) for the DE-20 (green), Falcon II (red) and K2 Summit (blue). The noise power spectra have been scaled to unity at the origin.

Fig. 3 shows both the measured noise power spectra for the detectors (scaled so that they are ~1 at zero spatial frequency) and the MTF results from Fig. 2 plotted as ${\mathrm{MTF}}^{2}$. The ratio of the ${\mathrm{MTF}}^{2}$ to the noise power spectra determines the behaviour of the DQE as a function of spatial frequency. Fig. 3 illustrates the contrasting behaviours expected for the DQE in the K2 Summit and Falcon II detectors. The noise power spectrum of the K2 Summit is essentially constant and so the behaviour of the DQE is determined by that of the ${\mathrm{MTF}}^{2}$. In contrast the noise power spectra of the Falcon II and the corresponding ${\mathrm{MTF}}^{2}$ track each other so that the DQE of the Falcon II is relatively constant as a function of spatial frequency.

**Fig. 4:**
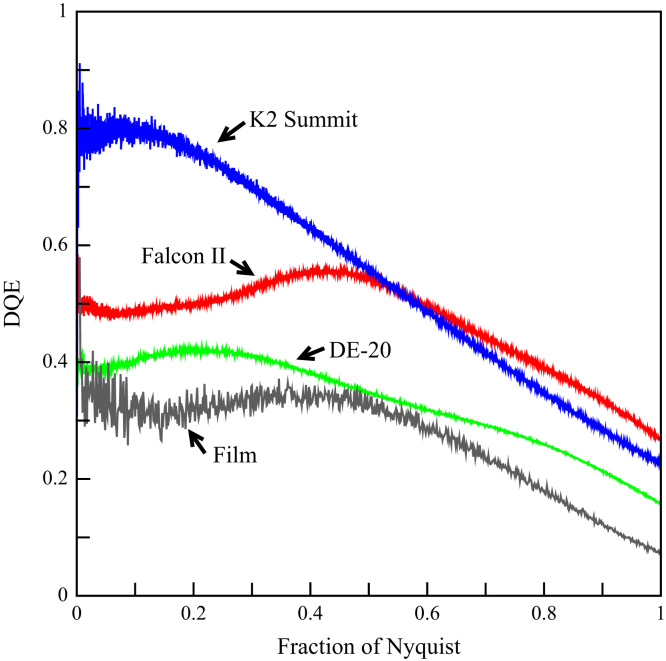
Measured DQE as a function of spatial frequency for the DE-20 (green), Falcon II (red) and K2 Summit (blue). The corresponding DQE of photographic film from [4] is shown in black.

Fig. 4 shows the DQE as a function of spatial frequency for the three detectors. For reference the corresponding DQE of Kodak SO-163 photographic film as given in [4] is also included. Clearly the counting mode of the K2 Summit gives it the highest DQE at low spatial frequencies. With increasing spatial frequency the DQE of the K2 Summit falls roughly as the MTF^2^ but those of the DE-20 and Falcon II stay fairly constant out to 1/2 the Nyquist frequency due to parallel falls in their respective noise power spectra. The fall in DQE towards the Nyquist frequency is expected due to increasing contributions from aliased terms in the noise power spectra. Beyond ~3/4 of the Nyquist frequency the DQE of the Falcon II becomes the highest of the three detectors. This occurs despite the Falcon II having the lowest MTF (the MTF at the Nyquist frequency of the K2 Summit in super-resolution mode is nearly 7 times that of the Falcon II).

**Fig. 5:**
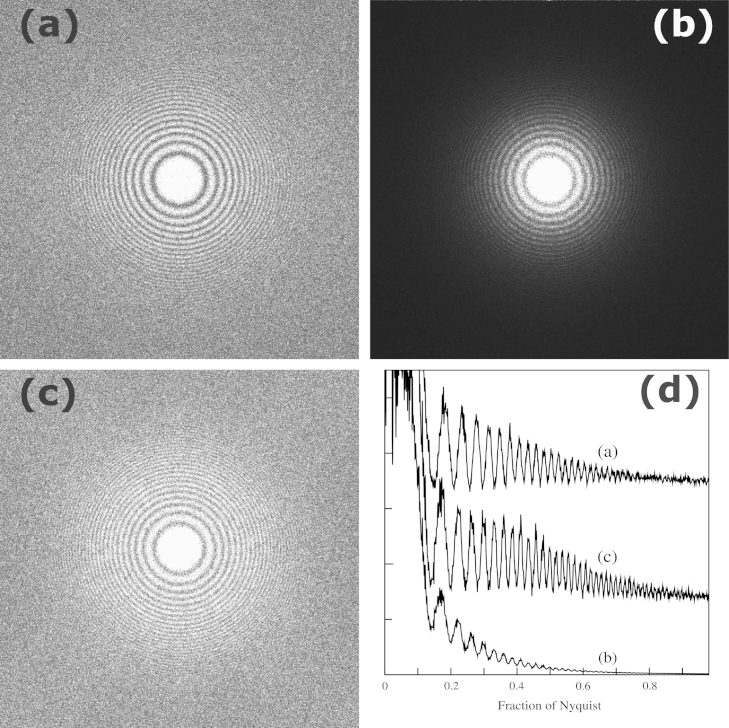
Comparison of the Thon rings seen in the power spectra of images taken of the same sample using the same number of electrons. The power spectra were obtained from a matching 1600×1600 area from the images. The power spectra from K2 Summit and Falcon II are shown in (a) and (b) respectively. The results in (c) show the results of (b) after they have been divided by the NNPS(ω) as described in the text. The circular averages over 90° of (a), (b) and (c) are shown in (d).

As the Falcon II and the K2 Summit are on the same microscope it was possible to compare directly their performance using an image of the same sample. To illustrate this we looked at the Thon rings from an amorphous carbon film. The different pixel sizes and positions of the cameras make it impossible to achieve exactly the same imaging conditions however the ~2.2 Å/pixel sampling obtained with the Falcon II in TEM mode and nominal magnification setting of SA37000 was within 5% of that obtained on the K2 Summit in EFTEM mode and nominal magnification setting of SA53000. The stability of the Krios column allows the same area of a sample to be seen with essentially the same defocus despite switching back and forth between EFTEM and TEM modes of the microscope. An area of carbon on a Quantifoil grid^5^ was first pre-irradiated for 5 min. A 30 s exposure at 3.5 e/pixel/s of part of this area was recorded in EFTEM mode using the K2 Summit and saved in 1 s blocks. The microscope was then switched to TEM mode and a long exposure of essentially the same area taken using the Falcon II in movie mode at an exposure rate of 0.66 e/pixel/frame. In order to match the exposures on both cameras the last 13 s of the K2 Summit image and the first 69 frames from the Falcon image were selected. The dose rates differ from those used to measure the DQE with that of the K2 Summit increased slightly so as to minimise drift while that of the Falcon II reduced to allow the total number of electrons in each exposure to be closely matched. The exposures from both detectors were drift corrected and a matching 1600×1600 area from both images selected. The power spectra from these are shown in Fig. 5. Despite having a total dose of only 45.5 electrons per pixel the Thon rings in both images go out to almost the Nyquist frequency.

The clarity with which the Thon rings in Fig. 5a can be seen is partly due to the fact that the underlying noise power spectra resulting from the counting mode of K2 Summit is essentially flat. It is not immediately obvious that the signal-to-noise ratio of the Thon rings shown in Fig. 5b from the Falcon II image is in fact very similar to that from the K2 at higher spatial frequency. In general, subtle variations and small signals, can easily be lost in among the rapidly changing background. To avoid this it is useful to mimic the K2 Summit behaviour and sharpen the images so that the underlying noise is flat. This requires dividing by a normalised noise power spectra, NNPS(ω) (or equivalently the square of the noise transfer function introduced in [9]). This is illustrated in Fig. 5c where the NNPS(ω) calculated from the measured MTF, as described in Appendix A, is used. The differences between the results for the K2 Summit in Fig. 5a and those of the Falcon II in Fig. 5c are difficult to see and so the circularly averaged values are given in Fig. 5d. For display purposes the plots have been arbitrarily displaced and as there was some residual astigmatism in the images the circular average was only carried out over 90°. As expected from the DQE results presented in Fig. 4 there is very little difference between the Thon ring visibility at high spatial frequencies from the two detectors.

## 4 Discussion

In the study of radiation sensitive samples the most important property in a detector is its DQE. The results presented here show that there are now three commercially available detectors that have higher DQE than photographic film. The low readout noise of these detectors also means that, unlike film with its finite fog level, their DQE remains high even at very low dose rates per image.

The observed differences in the MTF behaviour of the detectors will result in cosmetic differences between the resulting images. For example, images from the Falcon II will appear blurred relative to those obtained from a high MTF detector such as the K2 Summit. But as the MTF is known, the true signal strength incident on a detector can be in principle be recovered or simply sharpened as in Fig. 5c to better reflect the actual signal-to-noise. Provided a detector has a high DQE the fall in signal amplitude with increasing spatial frequency due to poor MTF will not affect the resolution that can be reached such as with a single particle reconstruction program like RELION [10].

The counting mode used by the K2 Summit, like that of the Medipix2 [11], eliminates readout noise. The DQE(0) is determined by the statistics of, and efficiency in, counting incident electrons and having no readout noise means that the K2 Summit can be used to arbitrarily low exposure rates without affecting the DQE. The readout noise in both the DE-20 and the Falcon-II is much lower than the average signal left by individual electrons and consequently will only start to reduce the value DQE(0) at very low exposure rates. The value of DQE(0) in Fig. 4 for both the DE-20 and the Falcon II is determined by the intrinsic variability in the energy deposited by incident electrons. Despite having a larger pixel size, the Falcon II MTF is lower than that of the DE-20. The Falcon II is therefore likely to have a thicker sensitive layer (the epilayer) leading to greater diffusion of charge carriers between pixels. Incident electrons will on average leave a greater signal in a thicker sensitive layer and the measured higher DQE of the Falcon II implies that the relative variance in this distribution is smaller, though this difference is difficult to see in Fig. 1d.

By using a counting mode, the K2 Summit is able to escape the intrinsic variability in the signal deposited by incident high energy electrons and achieve a higher DQE at low spatial frequency. Obtaining the maximum performance from a counting detector requires minimising coincidence losses and despite the K2 Summit running at 400 fps the coincidence losses quickly mount up. At 1 e/pixel/s the loss is negligible but at 10 e/pixel/s the results in [12] and the analysis in Appendix B say that the initial DQE(0) will be down by over 12%. Obtaining maximum performance from the K2 Summit requires using longer exposures that will inevitably need some form of drift correction [13].

The extrapolated value of DQE(0) in Fig. 4 for the K2 Summit is much lower than would be expected purely from coincidence losses at 1.1 e/pixel/s. The many problems faced by counting detectors are illustrated in Fig. 1. Some single events are spread over several pixels while others resemble tracks that the counting algorithm can either reject, treat as a single event, or treat as multiple events. The distribution of event energies, as shown in Fig. 1d, means that some events will always be barely above the readout noise and for practical reasons the loss of a proportion of these has to be accepted. Despite careful dark and gain calibration there will also inevitably be a few “hot” pixels that produce much higher noise levels. For integrating detectors, such as the DE-20 or Falcon, this is unimportant as the signal due to the readout noise is only a small fraction of the average signal from one electron. With a counting detector, false counts from the noise have the same weight as incident electrons. If a threshold is set so as to avoid generating counts in these pixels then too many true events will be lost and for this reason images from counting detectors often contain erroneously high counts in some pixels. In [5] the so-called “hot” pixels were identified as their signal was only in one pixel while true signals from incident electrons always spread into neighbouring pixels.

The spatial frequency dependence of the DQE in electron counting implementation used in the K2 Summit is determined primarily by that of the ${\mathrm{MTF}}^{2}$. This is a consequence of the almost flat noise power spectra resulting from putting the signal from an electron into a single pixel. Getting the best performance from the K2 Summit therefore requires maximising the MTF and so using the super-resolution mode. The counting implementation as used in the K2 Summit is not the only way to process individual incident electron events in order to build up an image. In particular, an improved DQE at higher spatial frequency can be obtained through retaining more sub-pixel information by using a distribution with finite spatial extent centred on the inferred incident position instead of putting all the weight for an electron in a single pixel. The optimal method of processing electron event images will of course depend on the incident electron energy and the underlying properties of the detector. For example in [5] it was found that a better DQE at higher spatial frequencies could be obtained by treating an incident electron image as a probability distribution for the incident position of an electron on the detector. This approach worked well for that particular combination of detector and incident electron energy but does not necessarily work in general. In particular this approach is not suited to the case such as the Falcon II where the event distribution is dominated by the effects of carrier diffusion. In [14] it was argued that instead of the raw image being used as a probability distribution the image should first be processed to remove effects such as a known point spread function and the processed image then be used as a probability distribution.

The lack of readout noise and high DQE at lower spatial frequency means that K2 Summit is particularly well suited to use in tomography. The near linear increase in DQE of the K2 Summit with decreasing spatial frequency means that there is always an advantage in improved signal-to-noise from using higher magnification. This also results in a higher dose rate on the sample and so shorter exposures but at the price of a reduced field of view.

The DQE of both the DE-20 and Falcon II detectors is relatively flat between zero and 50% of the Nyquist frequency. Because of this there is no advantage with these detectors in binning pixels or going to higher magnification. As lower spatial frequency information in a sample tends to be more resistant to radiation damage, higher signal-to-noise for these frequencies can be obtained using higher dose. To make use of this and yet avoid degrading higher spatial frequency information, images must be acquired as movies with the spatial frequency information from the frames weighted by a dose dependent weighting such as that used in [15]. In cryoEM studies where the particles being studied can be aligned with relatively low spatial frequency information (~20 Å) there should therefore be very little difference between the resolution that can be attained with all three of the detectors. Higher DQE will however reduce the number of particles and so time and effort required to reach a given resolution. The lower DQE of the DE-20 will in part be compensated for by the decreased number of images needed with its greater field of view. The higher dose rate that can be used on the DE-20 also leads to shorter overall exposure times which is advantageous on microscopes with side-entry cold stages that are inherently less stable. For particles whose images cannot be easily oriented, such as smaller or featureless particles, there is however no substitute for the higher DQE available by using higher magnification with the K2 Summit.

While all the detectors studied here represent an improvement in both DQE and convenience of use over film, in the long run the combination of high DQE, low readout noise and ability to capture time series data may produce the greatest impact. For example it is now clear that one of the major reasons for the low amplitude in cryoEM of high spatial frequency information is not just radiation damage but the presence of substantial initial movement of samples as the beam is turned on. The ability provided by the new detectors to see and quickly diagnose this movement will hopefully lead to a way of reducing, or eliminating, it and so lead to a further leap in ease of use and resolution that can be obtained with cryoEM.

## Appendix A Noise power spectra and MTF

The MTF at spatial frequency, (*ω*), measured in terms of the Nyquist frequency can be expressed as [4]

(3) $$\mathrm{MTF}(\omega )=\mathrm{sinc}(\pi \omega /2)\;{\mathrm{MTF}}_{0}(\omega )$$

in which sinc(πω/2)=sin(πω/2)/(πω/2) is the pixel modulation factor and ${\mathrm{MTF}}_{0}(\omega )$ can be viewed as the Fourier transform of an intrinsic point spread function, PSF(r), of the detector. For convenience ${\mathrm{MTF}}_{0}(\omega )$ can be expanded as the normalised sum of Gaussian functions with different length scales, *λ*_k_, i.e.,

(4) $${\mathrm{MTF}}_{0}(\omega )=\underset{k}{\sum }a_{k}\;\exp(-\pi^{2}\lambda_{k}^{2}\omega^{2}/4)$$

in which the weights, *a*_k_, obey $\sum_{k}a_{k}=1$ and the values of *λ*_*k*_, and *a*_*k*_ are chosen to fit the measured edge spread function. The corresponding intrinsic point spread function is

(5) $${\mathrm{PSF}}_{0}(r)=\underset{k}{\sum }a_{k}\;\exp(-r^{2}/\lambda_{k}^{2})/\pi \lambda_{k}^{2}$$

The noise power spectrum, NPS(ω), of *n* randomly distributed electrons with response given by Eq. (5) is proportional to the modulus squared of the Fourier transform of this response. For a given pixel size the Fourier transform of Eq. (5) will contain terms that are beyond the Nyquist frequency. These terms are aliased and contribute to the NPS(ω) [9]. Beyond 50% of the Nyquist frequency, non-circular terms need to be included the noise power spectra so that it becomes $\mathrm{NPS}(\omega_{x},\omega_{y})$ in which *ω*_*x*_ and *ω*_*y*_ are the fractions of the Nyquist frequency in the *x* and *y* directions. In the simplest case the additional non-circular terms arise from the pixel modulation term so that Eq. (3) becomes

(6) $$\mathrm{MTF}(\omega_{x},\omega_{y})=\mathrm{sinc}(\pi \omega_{x}/2)\;\mathrm{sinc}(\pi \omega_{y}/2)\;{\mathrm{MTF}}_{0}(\sqrt{\omega_{x}^{2}+\omega_{y}^{2}}).$$

and

(7) $$\mathrm{NNPS}(\omega_{x},\omega_{y})=\underset{i,j}{\sum }{\mathrm{MTF}}^{2}(\omega_{\mathrm{xi}},\omega_{\mathrm{yj}})$$

where the sum is over all aliased terms $\left(\omega_{\mathrm{xi}},\omega_{\mathrm{yj}})=(\omega_{x}+i,\omega_{y}+j\right)$ with i,j=0,±2,±4,… (in units of the Nyquist frequency). The results in Fig. 5c for the Falcon II were obtained by dividing the measured noise power spectra by the corresponding $\mathrm{NNPS}(\omega_{x},\omega_{y})$ calculated from the parameters *a*_*k*_ and *λ*_*k*_ used to fit the MTF.

## Appendix B Counting detector probabilities

An electron incident on a detector may not be detected at all, or can leave signal in one or more pixels. In general it is not possible to distinguish whether signal in adjacent pixels comes from one or more incident electrons and so a counting detector will simply record an electron arrival in response to events in which there is a signal in one or more adjacent pixels. This is correct in the limit with very few electrons incident per frame but with increasing numbers of electrons incident per frame observed events will increasingly result from two or more incident electrons. To examine the effects of this coincident loss we consider the simple case with in which there are on average *n* electrons per frame landing independently on a detector and these electrons are detected solely in one pixel. The probability that no electrons land on a pixel is given by exp(−n) with the corresponding probability that one or more electrons landing being given by 1−exp(−n). For a perfect counting detector the probability of an event being recorded in a single pixel, *p*_1_, is given by the product of the probability that one or more electrons land on a pixel multiplied by the probability that no electrons land on the surrounding 8 pixels. Similarly, the probability of a double event, *p*_2_, in which signal is recorded in adjacent pixels is given by the product of the probability for one or more electrons landing on two adjacent pixels and no electrons landing on the surrounding pixels. The adjacent pixels can be either horizontal/vertical or diagonal and have either 10 or 12 surrounding pixels respectively. Similarly for a 3 pixel event, *p*_3_, but now there are 20 ways for this to occur. In terms of *n* the probabilities for the events are given by

(8) $$p_{1}=e^{-8n}(1-e^{-n})$$

(9) $$p_{2}=(2e^{-10n}+2e^{-12n}){\left(1-e^{-n}\right)}^{2}$$

(10) $$p_{3}=(6e^{-12n}+8e^{-14n}+4e^{-15n}+2e^{-16n}){\left(1-e^{-n}\right)}^{3}.$$

For small *n*, the probability of recording an event is $p_{t}\approx p_{1}+p_{2}+p_{3}$ so that

(11) $$p_{t}=n-\frac{9}{2}n^{2}+\frac{49}{6}n^{3}+\cdots .$$

Counting electrons in this way leads to a very simple auto-correlation function with a value equal to *p*_*t*_ at the origin, a value of zero in pixels surrounding the origin and a value of p_*t*_^2^ everywhere else. From this the expected power spectra can be calculated. In units where the mean and variance of NPS for *n* random electrons is *n*, the values of the NPS at the origin (0,0), Nyquist frequency (1,0) and the corner (1,1) are $p_{t}-9p_{t}^{2}$, $p_{t}+3p_{t}^{2}$ and $p_{t}-p_{t}^{2}$ respectively. The simple model presented here therefore predicts the suppression of NPS at zero spatial frequency relative to that at the Nyquist frequency as seen experimentally with the K2 Summit [16], [12]. Note that the output noise at zero spatial frequency, given by $p_{t}-9p_{t}^{2}$, is less than the corresponding input noise value of *n*. The reduction in noise at zero spatial frequency does not lead to an increased DQE as the output signal is reduced. In particular at zero spatial frequency the signal is given by $dp_{t}/dn$, and using Eq. (11) gives

(12) $${\mathrm{DQE}}_{n}(0)={\left(\frac{dp_{t}}{dn}\right)}^{2}\frac{n}{p_{t}-9p_{t}^{2}}\approx 1-\frac{9}{2}n-\frac{239}{12}n^{2}.$$

From Eqs. (11), (12) a counting detector operated at *n*=0.025, such as the K2 Summit with 10 e/pixel/s, will have a 12% coincidence loss and a 14% drop in DQE(0).

## Appendix C Supplementary data

Application 1

## Footnotes

1 Direct Electron, LP, www.directelectron.com

2 FEI, www.fei.com

3 Gatan, Inc., www.gatan.com

4 SEM Probe Current Meter, part No. 087-001, www.deben.co.uk.

5 Quantifoil Micro Tools GmBH, www.quantifoil.com.
