# Supplementary material for: Comparison of optimal performance at 300 keV of three direct electron detectors for use in low dose electron microscopy
Source: Ultramicroscopy. 2014 Dec;147:156–63. doi: 10.1016/j.ultramic.2014.08.002 (PMC4199116; doi:10.1016/j.ultramic.2014.08.002)
Supplement: Application 1 [file mmc1.pdf]

Figure S1: Calibration of the flu-screen exposure meter readings on the microscopes versus the currents measured using the SEM current probe meter connected to the energy filter drift tube.

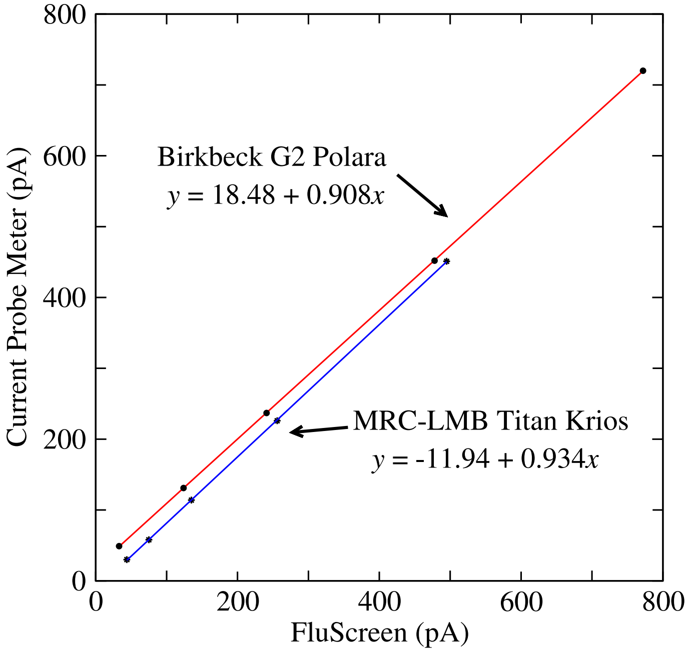

Figure S2: Measured edge spread functions, ESF, for the DE-20, Falcon II and K2 Summit. In each figure the measured ESF is in back, the multiple Gaussian fit in red and the difference between these in green. In terms of the fitted expansion parameters the Gaussian fit is given as[4]

$$\mathrm{ESF}(x) = \frac{1}{2}\sum_i \lambda_i\{\sum_{\sigma=\pm1}\sigma(t_\sigma/\lambda_i \mathrm{erfc}(-t_\sigma/\lambda_i) - \exp(-t_{i\sigma}^2/\lambda_i^2)/\sqrt{\pi})\}$$

in which x is measured in pixels and $t_\sigma = (x+\sigma)$.

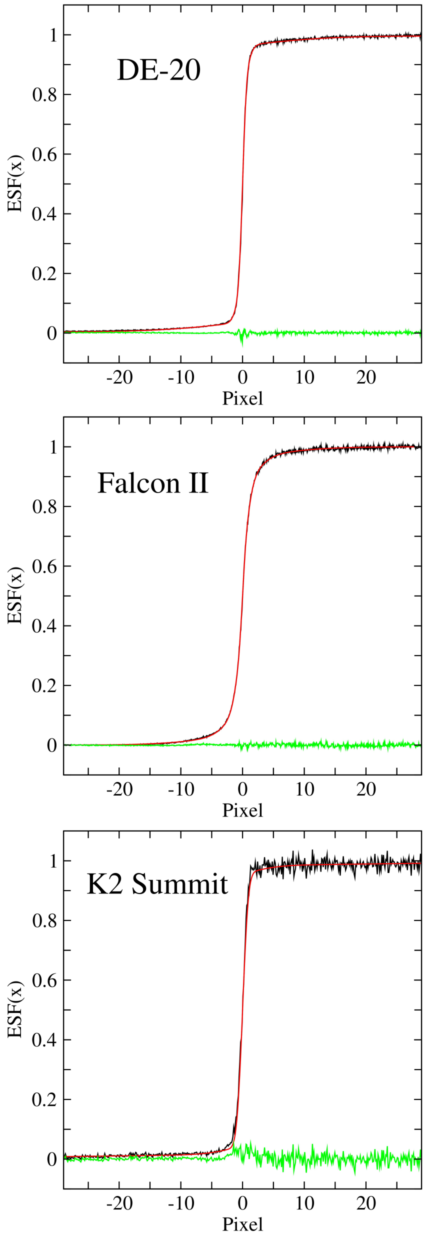

Table S1: Expansion parameters used with Eqns. (3, 4) to fit the measured MTF of the detectors. In particular $\mathrm{MTF}(\omega) = \mathrm{sinc}(\pi\omega/2)\sum_i a_i \exp(-\pi^2\lambda_i^2\omega^2/4)$.

Detector	a_i	λ_i
DE20	0.559358	0.487333
	0.367138	1.396542
	0.063836	15.203810
	0.009668	192.352501
Falcon II	0.530135	2.166934
	0.311621	0.674490
	0.138967	9.583019
	0.019277	49433.330392[1]
K2 Summit (SR)	0.910214	0.888312
	0.047163	4.467267
	0.042623	57.442789

[1] This term represents the long range scattering contribution such as from the camera housing. The large value of λ produces an initial drop in MTF around zero spatial frequency. The value of λ must be large but the actual value is not well defined.
